# All-Trans Retinoic Acid Inhibits Bone Marrow Mesenchymal Stem Cell Commitment to Adipocytes via Upregulating FRA1 Signaling

**DOI:** 10.1155/2020/6525787

**Published:** 2020-01-31

**Authors:** Linjun Xie, Liying Zou, Jie Chen, Youxue Liu

**Affiliations:** ^1^Children's Nutrition Research Center, Children's Hospital of Chongqing Medical University, Chongqing Key Laboratory of Child Nutrition and Health, Chongqing, China; ^2^Ministry of Education Key Laboratory of Child Development and Disorders, Chongqing, China; ^3^National Clinical Research Center for Child Health and Disorder, Chongqing, China; ^4^China International Science and Technology Cooperation Base of Child Development and Critical Disorders, Chongqing, China

## Abstract

Obesity, caused by an increased number and volume of adipocytes, is a global epidemic that seriously threatens human health. Bone marrow mesenchymal stem cells (BMSCs) can differentiate into adipocytes. All-trans retinoic acid (atRA, the active form of vitamin A) inhibits the adipogenic differentiation of BMSCs through its receptor RARG. The expression level of FRA1 (FOS like 1, AP-1 transcription factor subunit) in atRA-treated BMSCs increased, suggesting that atRA-mediated inhibition of BMSCs adipogenesis involves FRA1. BMSCs were transfected with adenovirus overexpressing *Fra1* (ad-fra1) or silenced for *Fra1* (si-fra1) and then treated with atRA. BMSCs treated with atRA and treated with ad-fra1 showed decreased mRNA and protein levels of key adipogenic genes (*Pparg2*, *Cebpa*) and adipogenesis-associated genes (*Cd36*, *Fabp*, *Lpl*, and *Plin*); atRA had a stronger inhibitory effect on adipogenesis compared with that in the ad-fra1 group. Adipogenic gene expression in *Fra1*-silenced BMSCs was significantly upregulated. Compared with that in the atRA group, the si-fra1 + atRA also upregulated adipogenic gene expression. However, compared with si-fra1, si-fra1 + atRA significantly inhibited adipogenic differentiation. Chromatin immunoprecipitation showed that RARG directly regulates *Fra1* and FRA1 directly regulates *Pparg2* and *Cebpa*. The results supported the conclusion that atRA inhibits BMSC adipogenesis partially through the RARG-FRA1-PPARG2 or the CEBPA axis or both. Thus, vitamin A might be used to treat obesity and its related diseases.

## 1. Introduction

Currently, obesity is considered a major health hazard and a global epidemic. The World Health Organization reported that in 2016, there were over 19 billion adults who were overweight, and 6.5 billion adults were obese (http://www.who.int/topics/obesity/en/). Obesity is a major risk in many diseases, including type 2 diabetes, cardiovascular dysfunction, musculoskeletal disorders, Alzheimer's disease, and cancers (prostate, colorectal, endometrial, and breast) [1–3], even impairing quality of life and leading to depression [4]. Mesenchymal stem cells (MSCs) exist in many tissues and have the potential for multidirectional differentiation. They can differentiate into adipocytes, osteoblasts, chondrocytes, nerve cells, or cardiomyocytes [5]. Bone marrow-derived MSCs (BMSCs) are considered the main type of MSCs for laboratory research and clinical application [6]. The important pathophysiological mechanism of obesity is the increase in the number and volume of adipocytes. The increase in adipocytes is caused by the recruitment of new preadipocytes from MSCs [7]. The differentiation of MSCs is regulated by many factors, among which the most critical molecular interactions are the members of the CCAAT/enhancer binding protein (CEBP) and peroxisome proliferator-activated receptors (PPARs) families [8, 9]. Exploring the molecular mechanism of obesity is necessary to prevent and treat obesity and its related diseases.

Vitamin A is an important fat-soluble micronutrient with well-established roles in embryonic development, reproduction, neuronal cell growth, immune function, and vision [10–13]. All-trans retinoic acid (atRA) is an active form of vitamin A, a small amount of which can help the body stay healthy [14]. *In vitro* and *in vivo* studies have demonstrated atRA as a key regulator in adipose tissue metabolism and obesity [15–17]. Our previous study demonstrated that atRA could inhibit the adipogenic differentiation of BMSCs by downregulating the expression level of peroxisome proliferator-activated receptor gamma 2 (PPARG2) via its receptor retinoic acid receptor gamma (RARG) [18]. However, chromatin immunoprecipitation (ChIP) analysis confirmed that the RARG protein does not bind to the *Pparg2* promoter to directly regulate its expression [19]. How atRA plays its role in regulating adipogenic differentiation remains unclear; therefore, the present study aimed to research the underlying signals.

Activator protein-1 (AP1) is an important transcription factor family that regulates cell proliferation and differentiation, which consist of the FOS family (FOS, FOSB, FRA1, and FRA2) and JUN family (JUN, JUNB, and JUND) [20]. In recent years, some scholars have found that AP1 plays an important role in regulating adipocyte formation and osteoblast function. Hasenfuss et al. discovered that AP1 family members regulate the expression level of PPARG2 through the formation of homo or heterodimers, thereby affecting the lipid metabolism of hepatocytes [21]. Experiments *in vivo* demonstrated that *Fra1* overexpression (encoding FOS like 1, AP-1 transcription factor subunit) caused severe lipodystrophy in *Fra1* transgenic mice. In addition, previous research in our laboratory showed that the mRNA and protein expression levels of *Fra1* in BMSCs increased significantly after atRA intervention [19]. These observations suggested that FRA1 could be a key factor in atRA-induced inhibition of the adipogenic differentiation pathway. To clarify the potential mechanism, BMSCs were differentiated into adipocytes *in vitro* to explore the regulatory mechanism of FRA1 in atRA-induced inhibition of the adipocyte differentiation signaling pathway.

## 2. Materials and Methods

### 2.1. Plasmid Construct

The plasmids used in this study were designed to function in rat species. An adenovirus plasmid for *Fra1* overexpression (ad-fra1) and an adenovirus short hairpin RNA (shRNA) plasmid to silence *Fra1* (si-fra1) were designed and produced by Obio Technology Corp. (Shanghai, China). The *Fra1* gene sequence was synthesized and inserted into vector pAdeno-MCMV-3Flag-PA2-EGFP, to obtain adenovirus vector pAdeno-MCMV-Fra1-3Flag-PA2-EGFP ad-fra1. The si-fra1 shRNA sequences were inserted into vector pDKD-CMV-eGFP-U6, taking advantage of the AgeI and EcoRI enzymes to construct the shuttle plasmid and skeleton plasmid of the target gene in HEK293 cells. Three si-fra1 target sequences were used: Y7339 ATCCACTGCAATTCCTGGC, Y7340 TTCTTGTCTTCTTCTGGGA, and Y7341 TGCTACTCTTTCGATGGGC.

### 2.2. Cell Culture

BMSCs were obtained from the bone marrow of 2-week-old male Sprague-Dawley (SD) rats (*n* = 16, obtained from Chongqing Medical University Animal Care Centre, Chongqing, China) as described previously [22]. In detail, bone marrow was rinsed from femurs and tibia using culture medium that contained Dulbecco's modified Eagle's medium (DMEM)/F-12 (GIBCO, Grand Island, NY, USA), 10% fetal bovine serum (FBS) (AusGeneX, Brisbane, Australia), and 1% penicillin and streptomycin. BMSCs were obtained by whole bone marrow differential adherence method. In the first 24, 48, and 72 hours, unadhered cells could be effectively exchanged using total medium exchange. Thereafter, the medium was changed every 3 days. When the density of cells reached 95%, adherent BMSCs were digested with trypsin (GIBCO) and passaged on. Third-generation BMSCs were stored in a 90% FBS and 10% dimethyl sulfoxide (DMSO) (Dingguo Biotech, Beijing, China) mixture in liquid nitrogen. If necessary, BMSCs were resuscitated by rapid thawing in a 37°C water bath, plated in culture medium, and cultured at 37°C in humid air with 5% CO_2_. Third-generation BMSCs were used for all experiments [23]. BMSCs were maintained in 37°C and 5% CO_2_ incubators (Thermo Fisher Scientific, Waltham, MA, USA).

### 2.3. Multiplicity of Infection (MOI) Value Exploration and Efficiency of Adenovirus Transfection

BMSCs were seeded into a 24-well plate (6 × 10^5^ cells well^−1^). After overnight culture, the culture medium was exchanged and BMSCs were transduced with si-fra1 adenovirus (Y7340) at various MOI (100, 110, 120, 130, 150, 160, 180, and 200) at 37°C in a humidified 5% CO_2_, 95% air incubator for 12 h. The supernatant was then removed, fresh medium was added, and the cells were incubated for 72 h. The amount of fluorescence in the BMSCs was observed under a microscope (Nikon, Tokyo, Japan) under green fluorescence excitation. BMSCs were seeded into a 6-well plate (2 × 10^6^ cells well^−1^), the adenovirus (MOI 120) was into each well, grouped as follows: ad-fra1, si-fra1 (Y7339, Y7340, and Y7341) vector, and blank. The *Fra1* overexpression and knockdown efficiency were determined using quantitative real-time reverse transcription PCR (qRT-PCR).

### 2.4. In Vitro Transduction of BMSCs with Adenoviral Vector and Adipocyte Differentiation

BMSCs were seeded into a 6-well plate (2 × 10^6^ cells well^−1^); 6–8 h later, the medium was replaced at 2 ml per well. Adenoviral transient infection was carried out when the fusion degree of BMSCs reached more than 95%. The groups were set as follows: ad-fra1, si-fra1 (Y7340), vector + atRA, si-fra1 + atRA, and vector. Adenoviruses were added to the BMSCs and cultured at 37°C with 5% CO_2_ for 12 h. Thereafter, the BMSCs were washed with phosphate-buffered saline (PBS) (Dingguo Biotech, Beijing, China) and fed with adipogenic differentiation medium A (Cyagen, Jiangsu, China). In addition, atRA (Sigma, St. Louis, MO, USA), dissolved in pure ethanol, was added to vector + atRA and si-fra1 + atRA groups to achieve a concentration of 5 *µ*mol·L^−1^. Seventy-two hours later, we replaced medium A with fresh medium B and cultured the cells for 24 h. Fresh atRA was added to both medium A and B for the vector + atRA and si-fra1 + atRA groups. Medium A contained 87.5% BMSCs adipogenic differentiation basal medium A, 10% FBS, 1% penicillin-streptomycin, 1% glutamine, 0.2% insulin, 0.1% 3-isobutyl-1-methylxanthine (IBMX), 0.1% rosiglitazone, and 0.1% dexamethasone. Medium B (Cyagen) comprised 87.8% BMSCs adipogenic differentiation basal medium B, 10% FBS, 1% penicillin-streptomycin, 1% glutamine, and 0.2% insulin. In general, the adipogenic process comprised culturing BMSCs in medium A (2 ml per well) for 72 h. Old medium A was replaced with medium B (2 ml per well) for another 24 h. After that, the old medium B was replaced with fresh medium A at the same volume. This cycle was repeated three to five times until lipid droplets formed.

### 2.5. Oil Red O (ORO) Staining

BMSCs were fixed with 4% neutral formaldehyde for 30 min in room temperature on the 22^nd^ day of adipogenic induction and then washed with 1 × PBS twice. Oil red O dye solution (Cyagen) was formulated with 60% oil red O storage solution and 40% distilled water and filtered by neutral filter paper. Then, the BMSCs were stained with oil red O solution in a biosafety cabinet (Thermo Fisher Scientific, Waltham, MA, USA) for 60 min. After the oil red O dye solution was absorbed, the cells were washed three times with 1 ×  PBS. Next, adipogenic staining was observed under a microscope (*n* = 3 per group). Finally, the oil red O stained images were analyzed using ImageJ software (National Institutes of Health, Bethesda, MD, USA), following the methods described by Xia et al. [24].

### 2.6. RNA Isolation and qRT-PCR

Total RNA was extracted from cells using an RNA-Quick Purification kit (YiShan Biotech, Shanghai, China) according to manufacturer's instructions. The RNA concentration was measured using spectrophotometer (Nanodrop Technologies, Wilmington, DE, USA). Then reverse transcription of RNA into cDNA was performed following the PrimeScript Buffer kit instructions. QRT-PCR analysis was performed using a thermocycler (Bio-Rad, Hercules, CA, USA) and SYBR-Green Realtime-PCR Kit (Takara, Shiga, Japan) according to the manufacturer's instructions (*n* = 7 per group). The primer sequences used in this experiment are shown in Table 1, and *Gapdh* served as a control gene. All primers were synthesized by Huada Gene Company (Shenzhen, China). Fold-changes were compared after standardization with *Gapdh* and calculated using the 2^−ΔΔCt^ method [25].

### 2.7. Isolation of Total Protein and Western Blotting

We followed the methods of Lai et al. [26]. The radio immunoprecipitation assay (RIPA) lysis buffer (KeyGEN Biotech, Nanjing, China) which contained 0.1% protease inhibitor cocktail (KeyGEN) was used to extract total protein from BMSCs. The concentration of proteins was determined using a BCA kit according to the manufacturer's instructions (ATGene, Chongqing, China) and a microtiter plate reader (Thermo Fisher Scientific). 5 × SDS-PAGE sample buffer was added to equal amounts of protein, which were boiled at 100°C in a water bath for 10 min before being subjected to 8% SDS-PAGE.

Western blotting was performed as previously described [27]. Some adjustments were made, we used anti-RARG (Abcam, Cambridge, UK) (1 : 1000), anti-FRA1 (Abcam) (1 : 100), anti-CEBPA (Abcam) (1 : 1000), and anti-PPARG2 (Abcam) (1 : 500) antibodies according to the manufacturer's instructions (*n* = 6 per group). An ECL Prime kit (Millipore Corp, Billerica, MA, USA) and an ECL Imaging system (Syngene G : BOX, Cambridge, UK) were used to analyze the levels of immunoreactive proteins. ImageJ software was used to analysis the intensity of the protein bands.

### 2.8. Chromatin Immunoprecipitation (ChIP)

ChIP was performed using a Millipore company a ChIP kit (EZ-ChIP, Millipore Corp) following its manufacturer's protocol and according to the details from Lai's report [26]. In brief, BMSC chromatin was sheared by sonication (medium power, 20 cycles of 30 s between pulses) to 200–1000 bp. The chromatin was incubated overnight at 4°C with primary antibodies recognizing FRA1 (Santa Cruz, CA, USA) or RARG (Santa Cruz) or IgG antibody (the negative control). QRT-PCR was carried out using the SYBR-Green mix and a Bio-Rad CFX Connect Teal-Time system (Bio-Rad) (*n* = 6 per group). Primers for the rat *Fra1*, *Pparg2*, and *Cebpa* genes were as follows: *Fra1* primer sense TCAGGAGTTCAAGGCCAGTC, antisense 5′-CTCTGGAAGGAGGTGTGAGG-3′; *Pparg2* primer sense 5′-CACTGGGAAGTTGGAGAAGGAA-3′, antisense 5′-TCTGGGGATTTGTGATGTTGAA-3′; *Cebpa* primer sense 5′-ATAAAGACGCACAATCTCAGCACTCT-3′, anti-sense 5′-GTCACCCACTTCCAGCCAACC-3′. The qRT-PCR samples were subjected to 1% agarose gel electrophoresis to confirm and detect the amplified fragments under a UV light (Syngene G:BOX, Cambridge, UK) [28]. Fold enrichment was calculated over IgG using 2^–ΔΔCT^, where ΔΔCT = (normalized Ct_ip_−Ct_IgG_).

### 2.9. Statistical Analysis

All data were represented by the mean ± SEM. GraphPad Prism 5.0 software (GraphPad Software, Inc. La Jolla, CA, USA) was used for this analysis. The data from the oil red O staining, qRT-PCR, western blotting, and ChIP-qPCR were analyzed using an unpaired Student's *t* test (two sets of data) or one-way analysis of variance (ANOVA) (>2 sets of data) as data. Adjusted *p* values were calculated using Tukey's HSD post hoc analyses. The significance level was set at *p* < 0.05.

## 3. Results

### 3.1. Efficiency of Adenovirus Transfection into BMSCs

BMSCs were transduced with adenoviral si-fra1 (Y7339), which also expressed the enhanced green fluorescent protein (EGFP). The intracellular fluorescence intensity was used to evaluate transfection efficiency of adenovirus. Si-fra1 was infected at a MOI 100, 110, 120, 130, 150, 160, 180, and 200, and the intracellular fluorescence intensities were detected after 72 h of adenovirus transfection. The transfection efficiency, as determined by percentage of BMSCs containing green fluorescence, were 8.2%, 18.2%, 51.4%, 58.8%, 59.3%, 56.1%, 52.7%, and 66.1% at MOI of 100, 110, 120, 130, 150, 160, 180, and 200, respectively. There was a significant difference between the result for a MOI 120 and that for 110. However, above a MOI 120, the difference was not significant. Therefore, we chose adenovirus at a MOI of 120 for subsequent experiments.

Expression of untransduced (blank), vector, ad-fra1, and si-fra1 (Y7339, Y7340, and Y7341) mRNA was detected using qRT-PCR to confirm the level of *Fra1* mRNA expression. As expected, compared with the blank and vector, adenovirus ad-fra1 upregulated *Fra1* mRNA expression (*p* < 0.001), and adenovirus si-fra1 could markedly reduce the expression of the *Fra1* mRNA (*p* < 0.001). Y7340 had the most significant silencing effect and thus was chosen for future experiments. *Fra1* expression was not significantly different between the blank and vector. Figures are shown in Supplementary [Supplementary-material supplementary-material-1]. These data suggested that the *Fra1* gene overexpression and silencing were successful. In addition, the adenoviral vector itself would not affect the expression level of *Fra1*. In the latter experiment, we used the vector as the control group and the *Fra1* expression levels were maintained over 3 weeks.

### 3.2. AtRA Treatment Could Upregulate the Expression of RARg during the Adipogenic Differentiation of BMSCs

**Figure 1:**
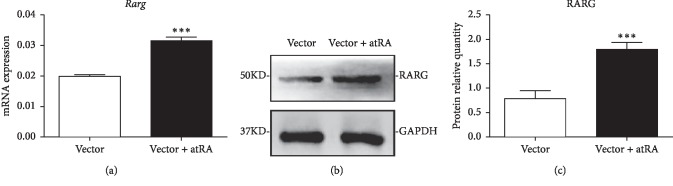
BMSCs treated with vector and vector + atRA for adipogenic differentiation for 8 days. (a) The mRNA expression of the target gene (*Rarg*), (*n* = 7/group). (b, c) The protein levels of RARG in the late stage of BMSC adipogenic differentiation (*n* = 6/group). The values are the mean ± SEM. ^*∗∗∗*^*p* < 0.001. BMSC, bone marrow mesenchymal stem cell; RARG, retinoic acid receptor gamma.

AtRA binds to three retinoic acid nuclear receptors (RAR*α*/*β*/*γ*) via noncovalent bonds. RAR*γ* plays an important role in the retinoic acid signaling pathway [18]. BMSCs were treated with 5 *μ*mol/L atRA and then induced for adipogenesis according to the previously mentioned method. After 8 days of treatment, qRT-PCR was used to analyze the expression of *Rarg* in the mRNA level. Using the vector group as the control group, *Rarg* mRNA expression was significantly increased in the vector + atRA group (*p* < 0.001) (Figure 1(a)). Western blotting showed the same results for the RARG protein level (*p* < 0.001) (Figures 1(b) and 1(c)). These data showed that atRA could increase the expression of RARG at both the mRNA and protein levels.

### 3.3. Overexpression of Fra1 and atRA Treatment Could Block BMSC Differentiation into Adipocytes

**Figure 2:**
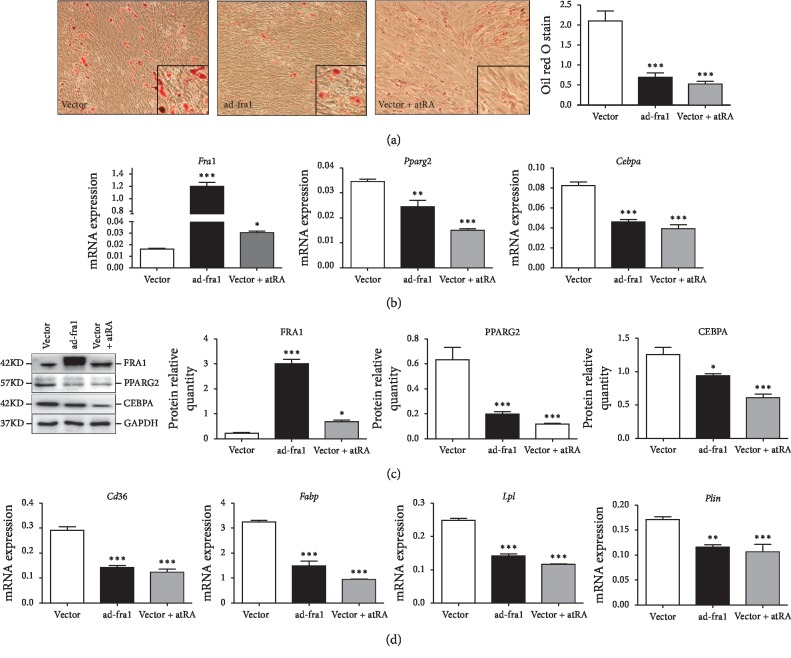
BMSCs treated with vector, ad-fra1, and vector + atRA for 22 days: (a) the effect of ad-fra1 and atRA on BMSCs adipogenic differentiation (oil red O staining ×100 and ×400), (*n* = 3/group). The mRNA expression in BMSCs treated with vector, ad-fra1, and vector + atRA for 8 days; (b) target gene (*Fra1*) and two adipocyte key genes (*Pparg2* and *Cebpa*); (d) adipogenesis-associated genes (*Cd36*, *Fabp*, *Lpl*, and *Plin*), (*n* = 7/group). (c) The protein levels of FRA1, PPARG2, and CEBPA in the late stage of BMSCs adipogenic differentiation (*n* = 6/group). The values are the mean ± SEM. ^*∗*^*p* < 0.05, ^*∗∗*^*p* < 0.01, ^*∗∗∗*^*p* < 0.001, ns = not significant in multiple comparisons. Both the ad-fra1 group and the vector + atRA group compared with the vector group. BMSC, bone marrow mesenchymal stem cell; FRA1, FOS like 1, AP-1 transcription factor subunit; atRA, all-trans retinoic acid; PPARG2, peroxisome proliferator-activated receptor gamma; CEBPA, CCAAT enhancer binding protein alpha; *Cd36*, CD36 molecule; *Fabp*, fatty acid binding protein; *Lpl*, lipoprotein lipase; *Plin*, perilipin.

The inhibitory effect of atRA on adipogenesis has been confirmed in many studies [18, 19, 29, 30]. We repeated a previous experiment with 5 *μ*mol/L atRA in BMSCs. After 22 days of adipogenic differentiation treatment, oil red O staining was performed to reveal the accumulation of fat-characterized adipocytes. The number of lipid droplets in the BMSCs treated with atRA was significantly fewer than those in the vector group (*p* < 0.001). Similarly, inhibition of differentiation to mature adipocytes and accumulation of lipid droplets were observed in the BMSCs overexpressing *Fra1* (*p* < 0.001), as indicated by a decreased number of Oil-Red-O-positive cells. ImageJ was used to analyze the pictures of Oil-Red-O staining, which was significantly inhibited compared with that in the vector only group (Figure 2(a)). The inhibition of BMSCs' adipogenic differentiation was stronger in the atRA group than in the ad-fra1 group.

PPARG is the motive force of adipocyte differentiation and comprises two different subsets, namely, PPARG1 and PPARG2. The expression of PPARG1 in adipocytes is very low, while PPARG2 has a high fat-selective function and is highly expressed in adipocytes [9]. When *Cebpa* was co-expressed with *Pparg2*, myoblastic cell lines also had the ability to convert to adipocytes [31]. Therefore, both *Pparg2* and *Cebpa* play important roles in adipogenesis. In addition, the induction of several genes characterizing functional mature adipocytes, namely, *Cd36* (CD36 molecule), *Fabp* (fatty acid binding protein), *Lpl* (lipoprotein lipase), and *Plin* (Perilipin) are adipogenesis-associated genes. Proteins and mRNA were extracted from BMSCs after 8 days of adipogenesis induction. QRT-PCR demonstrated markedly increased *Fra1* expression in the ad-fra1 group compared with that in the vector group (*p* < 0.001). Using the vector group as the control group, *Fra1* mRNA expression was increased in the vector + atRA group (*p* < 0.05). Conversely, both in the ad-fra1 and vector + atRA groups, the mRNA expression levels of *Pparg2*, *Cebpa* and the adipogenesis associated genes (*Cd36*, *Fabp*, *Lpl*, and *Plin*) decreased strongly (Figures 2(b) and 2(d)). Furthermore, the mRNA expression levels of these genes were even lower in the vector + atRA group. Western blotting showed the same results for FRA1, PPARG2, and CEBPA protein levels (Figure 2(c)). These data strongly indicated that FRA1 upregulation can partly imitate the effects of atRA and is associated negatively with BMSC adipocyte differentiation.

### 3.4. Knockdown of Fra1 In Vitro Promoted the Differentiation of BMSCs into Adipocytes

**Figure 3:**
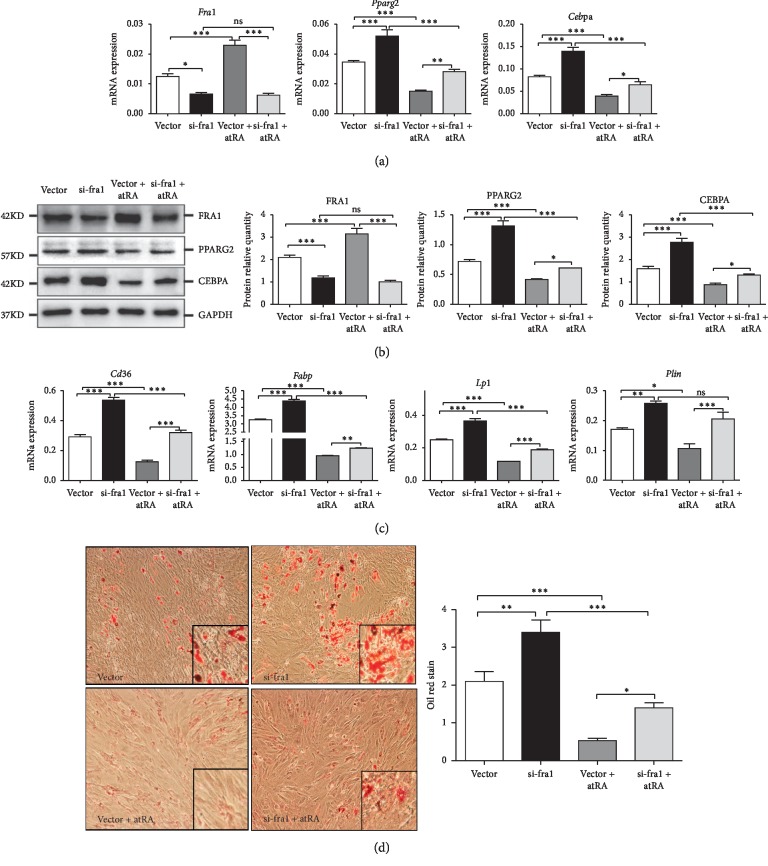
BMSCs treated with vector, si-fra1, vector + atRA, and si-fra1 + atRA for 8 days: (a) the mRNA expression of the target gene (*Fra1*) and two adipocyte key genes (*Pparg2* and *Cebpa*); (b) the protein levels of FRA1, PPARG2, and CEBPA in the late stage of BMSCs adipogenic differentiation (*n* = 6/group); (c) adipocyte-associated genes (*Cd36*, *Fabp*, *Lpl*, and *Plin*) (*n* = 7/group). BMSCs treated with vector, si-fra1, vector + atRA, and si-fra1 + atRA for 22 days; (d) the effect of ad-fra1 and atRA on BMSC adipogenic differentiation (oil red O staining ×100 and ×400) (*n* = 3/group). The values are the mean ± SEM. ^*∗*^*p* < 0.05, ^*∗∗*^*p* < 0.01, ^*∗∗∗*^*p* < 0.001, ns = not significant in multiple comparisons. The lines above the columns depict the compared groups. BMSC, bone marrow mesenchymal stem cell; FRA1, FOS like 1, AP-1 transcription factor subunit; atRA, all-trans retinoic acid; PPARG2, peroxisome proliferator activated receptor gamma; CEBPA, CCAAT enhancer binding protein alpha; *Cd36*, CD36 molecule; *Fabp*, fatty acid binding protein; *Lpl*, lipoprotein lipase; *Plin*, perilipin.

BMSCs were plated in 6-well plates at a density of 2 × 10^6^ cells per well. BMSCs were treated with adenoviruses silencing *Fra1* for 12 h, after which the cells were induced to differentiate to adipocytes for 8 days. QRT-PCR detected that the mRNA expression level of *Fra1* was significantly lower in si-fra1 group compared with that in the vector group (*p* < 0.01). The mRNA expression levels of *Cebpa* and *Pparg2* in the si-fra1 group were significantly upregulated compared with those in the vector group (*p* < 0.05 and *p* < 0.001, respectively) (Figure 3(a)). Western blotting showed similar results at the protein level (Figure 3(b)). The mRNA levels of adipocyte associated genes, which are markers of terminal adipogenesis, were upregulated in the si-fra1 group during adipogenesis differentiation 8^th^ day (Figure 3(c)). Compared with the vector group, si-fra1 group showed distinct lipid accumulation on the 22^nd^ day, as indicated by the amount of lipid droplets that could be seen using oil red O staining (Figure 3(d)).

Vector + atRA and si-fra1 + atRA groups were treated with empty adenoviral vector and the adenoviral vector silencing *Fra1* for 12 hours, after which they were treated with atRA and placed in adipogenic differentiation medium for 8 days. Using the vector group as the control, the *Fra1* mRNA was strongly silenced in si-fra1 + atRA group and there was no significant difference between si-fra1 group and si-fra1 + atRA group. QRT-PCR data showed that *Cebpa* and *Pparg2* expression levels were significantly upregulated in the si-fra1 + atRA group compared with those in the vector + atRA group (*p* < 0.01 and *p* < 0.05, respectively) (Figure 3(a)). Western blotting showed similar results for the protein levels (Figure 3(b)). The mRNA expression levels of the adipocyte associated genes were also significantly upregulated (Figure 3(c)). The increased adipogenesis was confirmed by oil red O staining (Figure 3(d)). Thus, the results confirmed that silencing *Fra1* promoted the differentiation of BMSCs into mature fat cells.

### 3.5. RARG Upregulates Transcription of Fra1 and FRA1 Downregulates the Transcription of Pparg2 or Cebpa by Binding to its Proximal Promoter after atRA Treatment

**Figure 4:**
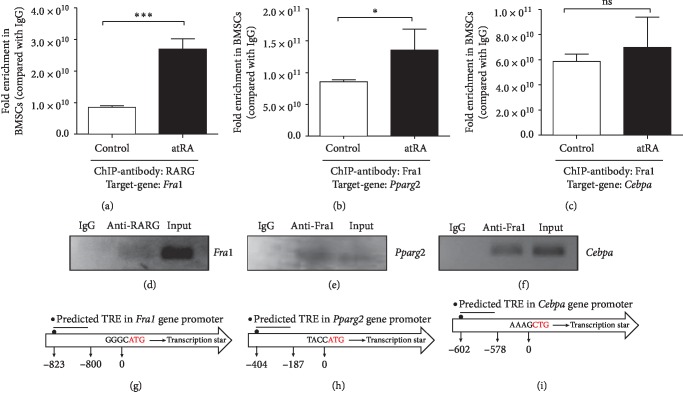
ChIP-qPCR and agarose gel electrophoresis detection of the interaction between (a, d) RARG and *Fra1*, (b, e) FRA1 and *Pparg2*, and (c, f) FRA1 and *Cebpa* in the atRA-induced inhibition of BMSCs adipogenic differentiation. The predicted binding sites of RARG protein in (g) the *Fra1* gene and (h) the FRA1 protein in (i) the *Pparg2* gene and *Cebpa* gene. Mean ± SEM (*n* = 6/group) ^*∗*^*p* < 0.05, ^*∗∗∗*^*p* < 0.001, ns = not significant in multiple comparisons. The lines above the columns depict the compared groups. BMSC, bone marrow mesenchymal stem cell; FRA1, FOS like 1, AP-1 transcription factor subunit; atRA, all-trans retinoic acid; PPARG2, peroxisome proliferator-activated receptor gamma; CEBPA, CCAAT enhancer binding protein alpha.

Previous research in our laboratory identified that atRA inhibits BMSC adipogenic differentiation by activating RARG [18]. The data in the present study showed that both atRA and FRA1 can downregulate the expression of adipocyte-associated genes. To further investigate the interaction between RARG and *Fra1*, we conducted a ChIP-qPCR experiment using anti-RARG antibodies. The ChIP-qPCR analysis clearly showed that the RARG protein was present on the *Fra1* promoter in BMSCs, with statistically significant enrichment after atRA treatment (*p* < 0.001) (Figure 4(a)). Agarose gel electrophoresis detection confirmed this finding (Figure 4(d)). The Genomatix software predicted that the RARG binding site in the promoter of *Fra1* is in the following sequence: TCCATCTCAATTGACCTTCCTCCAC. This site is 823 bp 5′ to the transcription initiation site (Figure 4(g)). Therefore, we concluded that RARG enters the nucleus after treatment with atRA to regulate *Fra1* expression by binding to its promoter region.

Our experiments showed that overexpression of *Fra1* downregulated *Pparg2* and *Cebpa* expression. Conversely, *Fra1* silencing upregulated *Pparg2* and *Cebpa* expression. To clarify the relationship between FRA1 and *Pparg2* and *Cebpa,* we conducted ChIP-qPCR tests using anti-FRA1 antibodies. The results showed that FRA1 protein was present at the *Pparg2* promoter in BMSCs and was significantly enriched after atRA treatment (*p* < 0.05) (Figure 4(b)). Agarose gel electrophoresis showed the same result (Figure 4(e)). The Promo software predicted that the FRA1 bind site in the promoter of *Pparg2* is in the following sequence: AGATGACTCAAAG. This site is 404 bp 5′ to the transcription initiation site (Figure 4(h)). The ChIP-qPCR data also showed that FRA1 protein was present at the *Cebpa* promoter in BMSCs; however, there was no statistically significant enrichment after atRA treatment (Figure 4(c)). Agarose gel electrophoresis showed that FRA1 was obviously enriched in the *Cebpa* sample (Figure 4(f)). The Genomatix software predicted a FRA1 binding site in the *Cebpa* promoter in the following sequence: AGACGCACAATCTCAGCACTCTGGG. This site is 602 bp 5′ to the transcription initiation site (Figure 4(i)). Overall, we confirmed that FRA1 enters the nucleus after treatment with atRA, where it regulates *Pparg2* and *Cebpa* expression by binding to their promoter regions.

## 4. Discussion

Vitamin A was the first identified fat-soluble vitamin. Its physiological functions include maintaining visual function [32], promoting immunoglobulin synthesis [33], and maintaining the stability of reproductive epithelial cells [34]. Recent research has focused on the physiological functions of vitamin A, and its metabolite atRA, such as regulating energy metabolism, insulin sensitivity, and lipid metabolism [35, 36]. An epidemiological investigation reported that a low serum retinol concentration was a risk factor for obesity. According to the data of the Third American Health and Nutrition Survey, the level of serum beta-carotene in obese people (P_95_ < BMI) was significantly lower than that in the normal weight group [37]. A similar survey was performed by Aasheim and his colleagues. They compared the serum levels of vitamin A in 110 patients with severe obesity with that in 58 healthy people and found that the levels of vitamin A in obese patients were significantly lower than those in the healthy controls [38]. Studies found that feeding fat rats with a vitamin A-supplemented diet could correct insulin resistance, plasma high-density lipoprotein level, and hypercholesterolemia in obese rats [39, 40]. In addition, the role of retinoic acid in inhibiting adipogenesis *in vitro* was confirmed in 3T3-L1 cells [41]. These studies demonstrated that vitamin A and atRA play important roles in inhibiting adipocyte differentiation, which provided a new focus for the clinical prevention and treatment of obesity.

BMSCs can be directed to differentiate into mature adipocytes *in vitro*. Our previous work found that atRA has a significant potential to inhibit the BMSCs' differentiation into adipocytes [18]. During this process, the level of RARG is upregulated, while the level of PPARG2 is downregulated. Co-IP analysis proved the connection between RARG and PPARG2 [18]. However, the result of ChIP-qPCR demonstrated that RARG could not regulate *Pparg2* directly [19]. The results of the present study reconfirmed the potential of adipogenic differentiation of BMSCs and the significant inhibition of atRA such adipogenic differentiations. Oil red O staining showed that the number of fat droplets in BMSCs after atRA treatment was significantly less than that in the control group. After atRA treatment, the expression levels of *Pparg2* and *Cebpa* and adipocyte associated genes decreased significantly.

FRA1 is a member of the AP-1 family. A previous study found that *Fra1* mRNA was highly expressed in the atRA intervention group [19]. Luther et al. showed that elevated FRA1 expression in mice can cause severe lipodystrophy [42]. Therefore, we aimed to determine how atRA inhibited BMSCs adipogenic differentiation through FRA1. BMSCs overexpressing *Fra1* showed few lipid droplets after oil red O staining and showed a significant decrease in adipogenic gene expression. The results were validated at the mRNA and protein levels. By contrast, BMSCs silenced for *Fra1* showed increased amounts of lipid droplets after oil red O staining. Furthermore, key adipogenic genes and adipogenesis-related genes showed higher expression in the si-fra1 group. This confirmed that FRA1 has an inhibitory effect on the induced adipogenic differentiation of BMSCs.

Although both atRA and FRA1 play a negative role in BMSCs' adipogenic differentiation, they have different efficiencies. Both ad-fra1 and atRA could increase the expression of *Fra1*. The mRNA and protein levels of *Fra1* in the ad-fra1 group were almost 40 times and 10 times higher, respectively, than those in the atRA intervention group. However, treatment with atRA caused stronger inhibition of adipogenesis. This was specifically reflected in the expression of key adipogenic genes (*Pparg2* and *Cebpa*) and adipocyte-associated genes. The PPARG2 and CEBPA protein levels also confirmed this result. In addition, after oil red O staining, a small amount of lipid droplets could be seen in the ad-fra1 group, but there were almost no lipid droplets in the atRA group. We hypothesized that silencing *Fra1* could promote BMSCs' adipogenic differentiation. Results from qRT-PCR, western blotting, and oil red O staining supported this hypothesis. We also found that although there were no statistical differences in the mRNA expression of *Fra1* between si-fra1 group and the si-fra1 + atRA group. Because of the role of atRA, the adipogenic ability in BMSCs was further inhibited in the si-fra1 + atRA group.

AtRA induces bioactivity by activating its nuclear receptors, RARs (RAR*α*/*β*/*γ*), and retinoid *X* receptors (RXR*α*/*β*/*γ*), which combine as a heterodimer to regulate target genes by binding to specific binding elements [43]. However, the regulatory mechanism of atRA remains unclear. The activation of PPARG is related to the inhibition of E2F dimerization partner 1 (E2F/DP, also known as transcription factor Dp-1 (TFDP1)). E2F/DP is a key transcription factor that regulates the growth of many kinds of cells [31]. The expression of PPARG precedes the expression of CEBPA, and CEBPA can re-upregulate the expression of PPARG [31]. Based on our research results, we propose the hypothesis that atRA inhibits BMSCs' adipogenic differentiation via the atRA-RARG-FRA1-PPARG2/CEBPA axis. Before the ChIP-qPCR experiment, we used the software PROMO and Genomatix to predict the binding sites of transcription factors. Interestingly, five potential RAR binding sites were predicted in the promoter of the rat *Fra1* gene. ChIP-qPCR analysis identified an 823 bp 5′ to the transcription initiation site of the *Fra1* promoter that contained a unique RAR binding site, which suggested that this proximal RAR binding site would be sufficient to drive the repression of *Fra1* transcription by RARG. In addition, we analyzed the regulatory relationship between FRA1 and *Pparg2* in a ChIP-qPCR experiment. The results showed that the DNA precipitated by the anti-FRA1 antibodies could amplify the promoter region sequence of *Pparg2*. These results indicated that FRA1 could directly regulate *Pparg2* expression by binding to its promoter region. Hasenfuss et al.'s findings also supported this result [21]. D'Ambrosio et al. used human hepatocellular carcinoma cells (HuH7) and human renal epithelial cells (293T) to detect luciferase reporter genes, which showed that FRA1 could significantly inhibit *Pparg2* gene transcriptional activity [17]. We used anti-FRA1 antibodies to precipitate *Cebpa* gene fragments and found that FRA1 was present at the *Cebpa* promoter in BMSCs. Similarly, Luther et al. detected FRA1 could directly inhibit *Cebpa* transcription [42].

**Figure 5:**
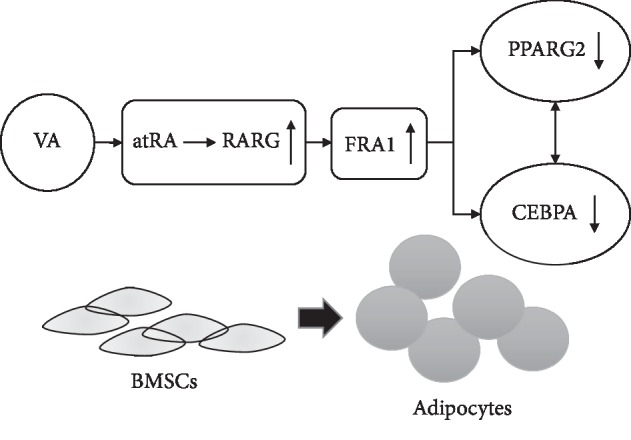
Mechanism by which vitamin A (VA) inhibits BMSCs' adipogenic differentiation via the atRA-RARG-FRA1-PPARG2/CEBPA axis. All-trans retinoic acid (atRA) is an active form of vitamin A, which acts on its nuclear receptor, RARG, and inhibits the expression of PPARG2 and/or CEBPA by upregulating FRA1, thereby inhibiting the differentiation of BMSCs into adipocytes. VA, vitamin A; atRA, all-trans retinoic acid; RARG, retinoic acid receptor gamma. FRA1, FOS like 1, AP-1 transcription factor subunit; PPARG2, peroxisome proliferator activated receptor gamma; CEBPA, CCAAT enhancer binding protein alpha; BMSC, bone marrow mesenchymal stem cell.

Our data showed that *Fra1* expression was increased in both the vector + atRA group and the ad-fra1 group, compared with that in the vector group. However, compared with that in the vector + atRA group, the expression of *Fra1* in the ad-fra1 group was higher. For the key adipogenic genes and adipocyte-associated genes, the vector + atRA group showed stronger expression inhibition than that in the ad-fra1 group. The result was also confirmed using western blotting and oil red O staining. QRT-PCR showed that in both the si-fra1 and si-fra1 + atRA groups, *Fra1* was silenced successfully and there was no statistical difference between them. However, the expression of master adipogenic genes, transcription factors, and lipogenic genes were statistically significantly decreased compared with that without atRA treatment, indicating that atRA reduced adipogenesis. Western blotting and oil red O staining confirmed this result. The ChIP-qPCR data proved that atRA could inhibit adipogenic differentiation of BMSCs through the RARG-FRA1-PPARG2 and/or CEBPA axis.  Figure 5 shows the inferred relationship between atRA, RARG, and FRA1, and two important downstream molecules PPARG2 and CEBPA.

Compared with the effect of FRA1 alone, atRA has a stronger inhibitory effect on adipogenesis. This suggested that atRA could inhibit adipogenesis via other pathways. Studies have shown that retinoic acid can stimulate the expression of transforming growth factor *β* (TGF*β*) effector protein SMAD family member 3 (SMAD3). SMAD3 interacts with CCAAT enhancer binding protein beta (CEBPB) to interfere with the binding of CEBP DNA to other transcription factors, thereby interfering with the adipogenic signaling pathway [44]. Ayala-Sumuano et al. demonstrated that retinoic acid inhibits 3T3-F442A cell adipogenesis by modulating CEBPB phosphorylation and downregulating sterol regulatory element binding transcription factor 1 alpha (SREBF1A) expression [45]. In addition, studies have shown that retinoic acid inhibits adipogenic differentiation of preadipocytes *in vitro* by upregulating the gene expression of transcription factors preadipocyte factor 1 (PREF1), SRY-box 9 (SOX9), and Kruppel-Like Factor 2 (KLF2) by activating RARG receptors. Retinoic acid receptor response elements (RAREs) are present in the promoter regions of these transcription factor genes [46–49]. Furthermore, Wang et al. discovered that RARG-C-FOS-PPARG2 signaling is critical for atRA-mediated inhibition of 3T3-L1 cell adipocyte differentiation [50]. Thus, atRA inhibits adipogenesis by affecting different signaling pathways, and the induction of atRA will have therapeutic potential in metabolic diseases such as obesity.

## 5. Conclusions

Taken together, our results show that atRA inhibits adipogenic differentiation of BMSCs by upregulating FRA1 signaling. We investigated FRA1 and atRA in the adipogenic differentiation of rat BMSCs. Both of them play a negative role in BMSC adipogenic differentiation. The molecular mechanism involves atRA exerting its effect via its nuclear receptor RARG and downregulating the expression of PPARG2 and CEBPA in the adipogenic differentiation of BMSCs by upregulating FRA1. In brief, the RARG-FRA1-PPARG2 or CEBPA axes, or both, are effective in the process of atRA inhibition of adipogenic differentiation of BMSCs. However, there might be other signaling pathways involved in atRA-mediated inhibition of BMSC adipogenesis, which should be explored in the future study. This deeper understanding of the molecular mechanism by which atRA inhibits BMSC adipogenesis supports the use of vitamin A to treat obesity and related diseases.

## Figures and Tables

**Table 1:** Specific primer sequences used for qRT-PCR analysis.

Target gene	Primer sequence (forward)	Primer sequence (reverse)
*Rarg*	5′-CTGACCCTGAACCGAACC-3′	5′-CACAGATGAGGCAGATAGCA-3′
*Fra1*	5′-TCCCAGAAGAAGACAAGAAGG-3′	5′-GGAGTCAGAGAGGGTGTGGT-3′
*Cebpa*	5′-GGTGGATAAGAACAGCAACGA-3′	5′-TCAACTCCAACACCTTCTGCT-3′
*Pparg2*	5′-CCTCCCTGAATAAAGATGG-3′	5′-CACAGCAAACTCAAACTTAGGC-3′
*Cd36*	5′-CTCTGACATTTGCAGGTCCA-3′	5′-AGTGGTTGTCTGGGTTCTGG-3′
*Fabp*	5′-AGCATCATAACCCTGGATGG-3′	5′-GCCTTTCATGACACATTCCA-3′
Lpl	5′-CTCGCTCTCAGATGCCCTAC-3′	5′-GCCACTGTGCCATACAGAGA-3′
*Plin*	5′-AGCTCCACTCCACTGTCCAT-3′	5′-CGTAGCCGACGATTCTCTTC-3′
*Gapdh*	5′-CCTGGAGAAACCTGCCAAG-3′	5′-CACAGGAGACAACCTGGTCC-3′

*Rarg*, retinoic acid receptor gamma; *Fra1*, FOS like 1, AP-1 transcription factor subunit; *Cebpa,* CCAAT enhancer binding protein alpha; *Pparg2*, peroxisome proliferator activated receptor gamma; *Cd36,* CD36 molecule; *Fabp*, fatty acid binding protein; *Lpl,* lipoprotein lipase; *Plin,* Perilipin; *Gapdh*, glyceraldehyde-3-phosphate dehydrogenase.

## Data Availability

The data used to support the findings of this study are available from the corresponding author upon request.

## Supplementary Materials

Supplementary Materials Supplementary Figure 1: the efficiency of adenovirus transfection into BMSCs. The optimal MOI for intervene BMSCs with adenovirus was 120. *Fra1* gene overexpression and silencing were successful in BMSCs.
