# Supplementary material for: All-Trans Retinoic Acid Inhibits Bone Marrow Mesenchymal Stem Cell Commitment to Adipocytes via Upregulating FRA1 Signaling
Source: Int J Endocrinol. 2020 Jan 31;2020:6525787. doi: 10.1155/2020/6525787 (PMC7013307; doi:10.1155/2020/6525787)
Supplement: Supplementary Materials — Supplementary Figure 1: the efficiency of adenovirus transfection into BMSCs. The optimal MOI for intervene BMSCs with adenovirus was 120. Fra1 gene overexpression and silencing were successful in BMSCs. [file 6525787.f1.pdf]

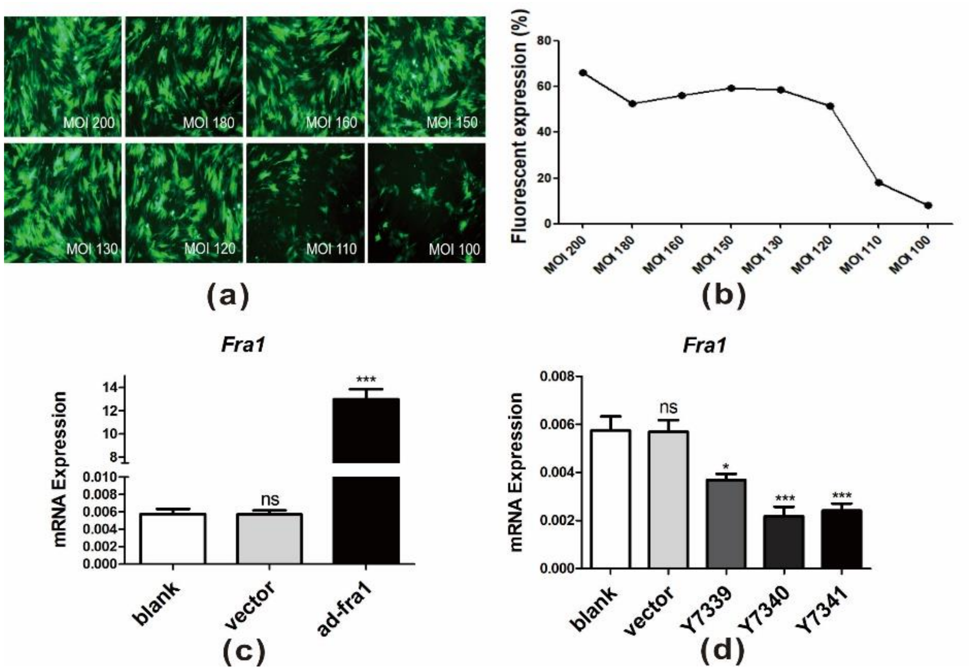


Supplementary Figure 1. Fluorescence images of BMSCs after 72 hours of intervention with Y7339 (a) in different MOI of adenoviruses (100, 110, 120, 130, 150, 160, 180, and 200) and (b) the fluorescence levels as analyzed by ImageJ. (c) *Fra1* mRNA levels in blank, vector, and ad-fra1 (d) and in blank, vector, and si-fra1 (Y7339, Y7340, and Y7341). The values show the mean ± SEM, (n = 7/group). The comparison of two data groups was analyzed using one-way ANOVA and P-values were calculated using Tukey's HSD post hoc analyses *$p < 0.05$, ***$p < 0.001$, ns = not significant in multiple comparisons. (c, d) Groups were compared with the blank group. BMSC, bone marrow mesenchymal stem cell; MOI, multiplicity of infection; FRA1, FOS like 1, AP-1 transcription factor subunit.
